# Robot-assisted anterior cruciate ligament reconstruction based on three-dimensional images

**DOI:** 10.1186/s13018-024-04732-w

**Published:** 2024-04-17

**Authors:** Gang Yang, Dingge Liu, Guangjin Zhou, Qining Wang, Xin Zhang

**Affiliations:** 1 Department of Sports Medicine, Peking University Third Hospital, Institute of Sports Medicine of Peking University, 49 North Garden Rd., Haidian District, Beijing, 100191 People’s Republic of China; 2 Beijing Key Laboratory of Sports Injuries, Beijing, China; 3 Engineering Research Center of Sports Trauma Treatment Technology and Devices, Ministry of Education, Beijing, China; 4 Department of Radiology, Peking University Third Hospital, Beijing, China; 5 Department of Advanced Manufacturing and Robotics, Peking University, Beijing, China; 6 Institute for Artificial Intelligence, Peking University, Beijing, China

**Keywords:** Anterior cruciate ligament reconstruction, Robot-assisted surgery, Arthroscopy, Tunnel placement

## Abstract

**Background **Tunnel placement is a key step in anterior cruciate ligament (ACL) reconstruction. The purpose of this study was to evaluate the accuracy of bone tunnel drilling in arthroscopic ACL reconstruction assisted by a three-dimensional (3D) image-based robot system.

**Methods **Robot-assisted ACL reconstruction was performed on twelve freshly frozen knee specimens. During the operation, three-dimensional images were used for ACL bone tunnel planning, and the robotic arm was used for navigation and drilling. Twelve patients who underwent traditional arthroscopic ACL reconstruction were included. 3D computed tomography was used to measure the actual position of the ACL bone tunnel and to evaluate the accuracy of the robotic and traditional ACL bone tunnel.

**Results **On the femoral side, the positions of robotic and traditional surgery tunnels were 29.3 ± 1.4% and 32.1 ± 3.9% in the deep-to-shallow direction of the lateral femoral condyle (*p* = 0.032), and 34.6 ± 1.2% and 21.2 ± 9.4% in the high-to-low direction (*p* < 0.001), respectively. On the tibial side, the positions of the robotic and traditional surgical tunnels were located at 48.4 ± 0.9% and 45.8 ± 2.8% of the medial-to-lateral diameter of the tibial plateau (*p* = 0.008), 38.1 ± 0.8% and 34.6 ± 6.0% of the anterior-to-posterior diameter (*p* = 0.071), respectively.

**Conclusions **In this study, ACL reconstruction was completed with the assistance of a robot arm and 3D images, and the robot was able to drill the bone tunnel more accurately than the traditional arthroscopic ACL reconstruction.

## Introduction

Anterior cruciate ligament (ACL) injury is a common injury, with an incidence of approximately 68.6 per 100 000 person-years and the rate of ACL reconstruction surgery is increasing [1]. Injuries of the ACL can cause instability of the knee joint, thereby increasing the risk of osteoarthritis [2]. In addition, ACL injuries were found to be associated with a sevenfold increase in the odds of total knee replacement [3]. Early surgery and postoperative rehabilitation are essential to restore the stability and function of the knee joint and prevent the occurrence and development of osteoarthritis [4–9]. ACL reconstruction is a procedure in which the surgeon inserts a graft through the femoral and tibial tunnels at the anatomical insertion of the ACL to reconstruct the native ACL fiber and restore stability to the knee joint [10–12]. A randomized controlled trial revealed better clinical outcomes in patients who underwent ACL reconstruction than in those who underwent rehabilitation [13].

For anatomic ACL reconstruction, accurate placement of the bone tunnel is a critical step in surgery. Tunnel malpositioning can lead to poor postoperative outcomes [14]. A tibial tunnel placed too far anteriorly in the tibial plateau increases the risk of graft impingement whereas if it is too far posteriorly, it increases the risk of rotational instability and worsens subjective outcomes [15, 16]. Moreover, femoral tunnel malpositioning is considered to be the most common technical error in primary ACL reconstruction [17].

Therefore, it is a great challenge for surgeons to easily and accurately place the bone tunnel always in the right position. Researchers have proposed a number of solutions to solve this problem. Computer-assisted navigation surgery was proposed for ACL reconstruction more than two decades ago and has shown good accuracy for the placement of bone tunnels, but its disadvantages are high cost and prolonged operation time [18, 19]. Intraoperative fluoroscopy was also used to assist with tunnel placement. However, it may not be practical in a real intraoperative setting to ensure optimal lateral positioning of the fluoroscope [20]. Robots are promising tools for orthopedic surgery and have been widely used in high-risk procedures such as spinal surgery. Compared with navigation-assisted surgery, robot-assisted surgery improved the accuracy of pedicle screw placement and reduced the duration of screw placement [21]. Previous studies have shown that the use of computer and robot-assisted techniques in ACL reconstruction surgery has the potential to reduce errors in tunnel placement [22–24]. In a previous study, knee bionic models were used to demonstrate that the robot's accuracy was greater than that of the traditional drilling method [25]. In this study, we performed ACL reconstruction surgery on fresh frozen human knee specimens using a three-dimensional (3D) image-based robotic system and compared it with traditional surgery to verify the accuracy of robot-assisted ACL surgery and to provide a theoretical basis for future practical application of robots in clinical practice.

## Materials and methods

### Study design

Our hospital Medical Science Research Ethics Committee approved this study (M2019056). The twelve fresh-frozen specimens used in this study were obtained from donations to our university anatomy program and included eight left knees and four right knees. Preoperative magnetic resonance imaging (MRI) was used to exclude specimens from patients with ligament injuries or cartilage diseases. In a simulated operating room, an experienced surgeon used a robotic system (TiRobot™, TINAVI Medical Technologies Co., Ltd., Beijing, China) combined with an arthroscopic system (Smith & Nephew, Inc., Arkansas, USA) to complete the registration of the ACL insertion points, the planning of the tunnels, and the drilling of the tunnels. Postoperative 3D computed tomography (CT) was used to measure the position and length of the actual tunnel and compare them with those of the planned tunnel. Twelve consecutive patients who underwent traditional arthroscopic ACL reconstruction performed by the same surgeon in our hospital were included. The ACL bone tunnel position was measured by 3D CT postoperatively and compared with the position of the bone tunnel drilled on the specimen by the robot. Finally, the bone tunnel positions of traditional surgery and robotic surgery were compared with the ACL positions reported in the literature to verify the anatomy of the bone tunnel positions of the two surgical methods.

### Robot preparation

**Fig. 1:**
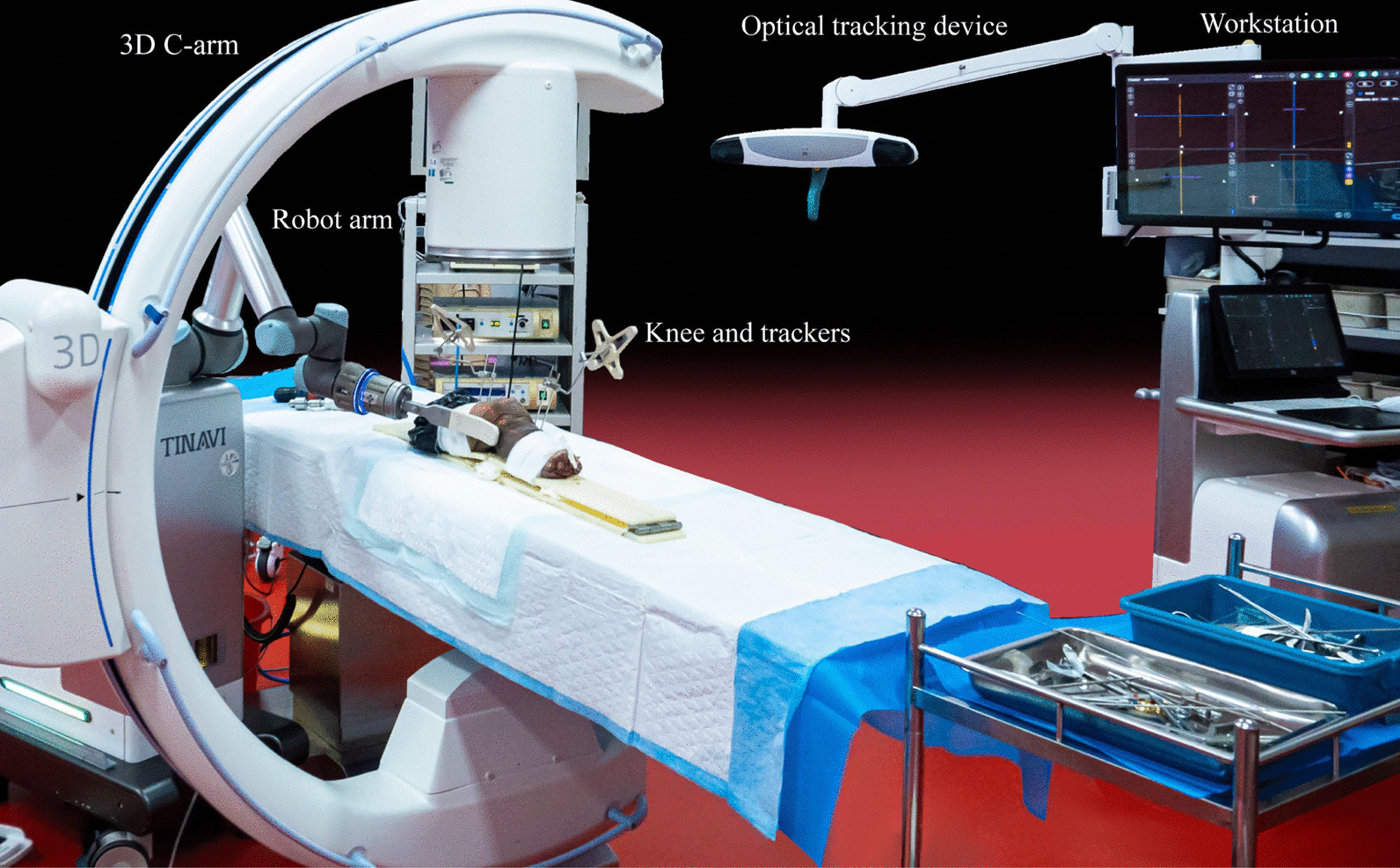
The appearance and placement of each part of the robot system

As shown in Fig. 1, the main part of the surgical robot system includes a robotic arm, a surgical planning and controlling workstation, and an optical tracking device. The surgical instruments associated with the robot system include two specially designed femoral and tibial locators for the cruciate ligament as well as femoral and tibial trackers.

The robot arm has 6 degrees of freedom and can perform automatic navigation and positioning flexibly. The workstation can plan and adjust the position of the bone tunnels according to the 3D images. The optical tracking device can track the spatial position of the patient's knee in real time. During surgery, the robot arm must be located on the same side as the surgeon, whereas the workstation and the optical tracking device must be located on the opposite side to prevent occlusion between the optical tracking device and the knee, which can affect the spatial positioning.

### Specimen preparation

Each specimen was thawed for 24 h before operation. The knee was positioned flex-90°, and standard anterolateral and anteromedial approaches were then established, and an arthroscope was used to observe the integrity of the intra-articular structures. Subsequently, the femoral and tibial trackers were fixed to the anterior of the femur and tibia, respectively, 15 cm from the knee joint line, respectively.

### Intraoperative planning

**Fig. 2:**
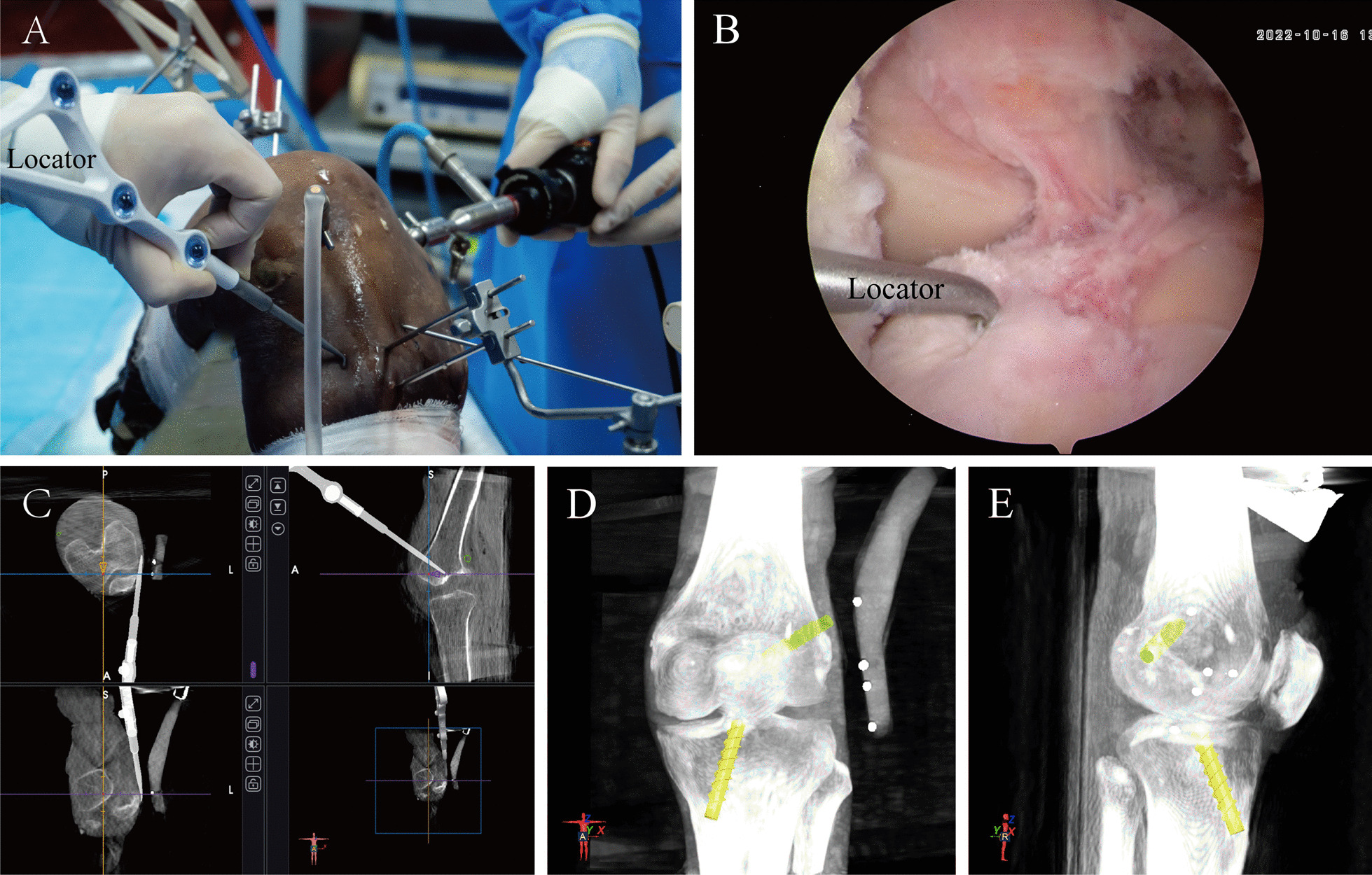
Preoperative planning process. **A** the tibial tunnel entry point planning process; **B** the tibial tunnel exit-point planning process; **C** simultaneous observation of the position of the locator in real time through imaging information provided by the doctors, in addition to the arthroscopic field of view and visual observation; **D** and **E** 3D views of the final generated femoral and tibial tunnels

After the trackers were fixed, the knee was straightened, placed within the robotic arm, and scanned with a 3D C-arm (Siemens, Munich, Germany). The 3D images generated after scanning were transmitted to the workstation of the robot to complete the registration of the knee and the images. The knee was subsequently placed on a rigid fixation frame with 90° flexion and arthroscopy was subsequently performed. The insertion point of the native ACL in the femur was registered by a specially designed ACL femoral locator via arthroscopy, and this insertion point was used as the exit point of the femoral tunnel. Similarly, the entrance to the femoral tunnel was registered on the lateral aspect of the lateral femoral condyle by the ACL femoral locator. The position of the locator can also be displayed on the workstation in real time when planning the tunnel (Fig. 2B). Once both exits and entrances were registered, the initial planned tunnel was automatically generated and viewed in a 3D image. At this point, the surgeon adjusted the position, angle, and length of the tunnel relative to the lateral femoral condyle according to the bony landmarks displayed in the 3D image and finally obtained a satisfactory femoral planning tunnel. Similarly, the tibial insertion of the ACL was selected as the exit of the tibial tunnel by the specially designed ACL tibial locator, and the appropriate position on the medial side of the tibia was selected as the entrance of the tibial tunnel (Fig. 2A). After the registration and tunnel adjustment, the planned tunnel of the tibia was also determined. Preoperative planning was completed after both the femoral and tibial tunnels were confirmed. The image above the workstation at this time is shown in Fig. 2C. The final planned bone tunnel generated on the 3D image is shown in Fig. 2D, E.

### Intraoperative ACL tunnel placement

The bone tunnels were drilled according to the planned tunnels generated during operation. In this process, the end of the robot arm and the trackers on the specimen must be in the field of view of the optical tracking device at all times so that the workstation can obtain the spatial position of the robot arm and the specimen in real time.

Drilling of the femoral tunnel was performed first. The ACL femoral tunnel was selected on the workstation, and the foot pedal of the robot arm was pressed to start the robot arm. During the movement of the robot arm, its position and the error between it and the planned tunnel are displayed in real time on the workstation. Finally, the robotic arm end automatically moved to the planned femoral tunnel entrance and stayed. The surgeon inserted a 2 mm Kirschner wire into the lateral femoral condyle through the guide sheath at the end of the robot arm and the ACL femoral tunnel was drilled along the Kirschner wire with an 8 mm femoral drill.

**Fig. 3:**
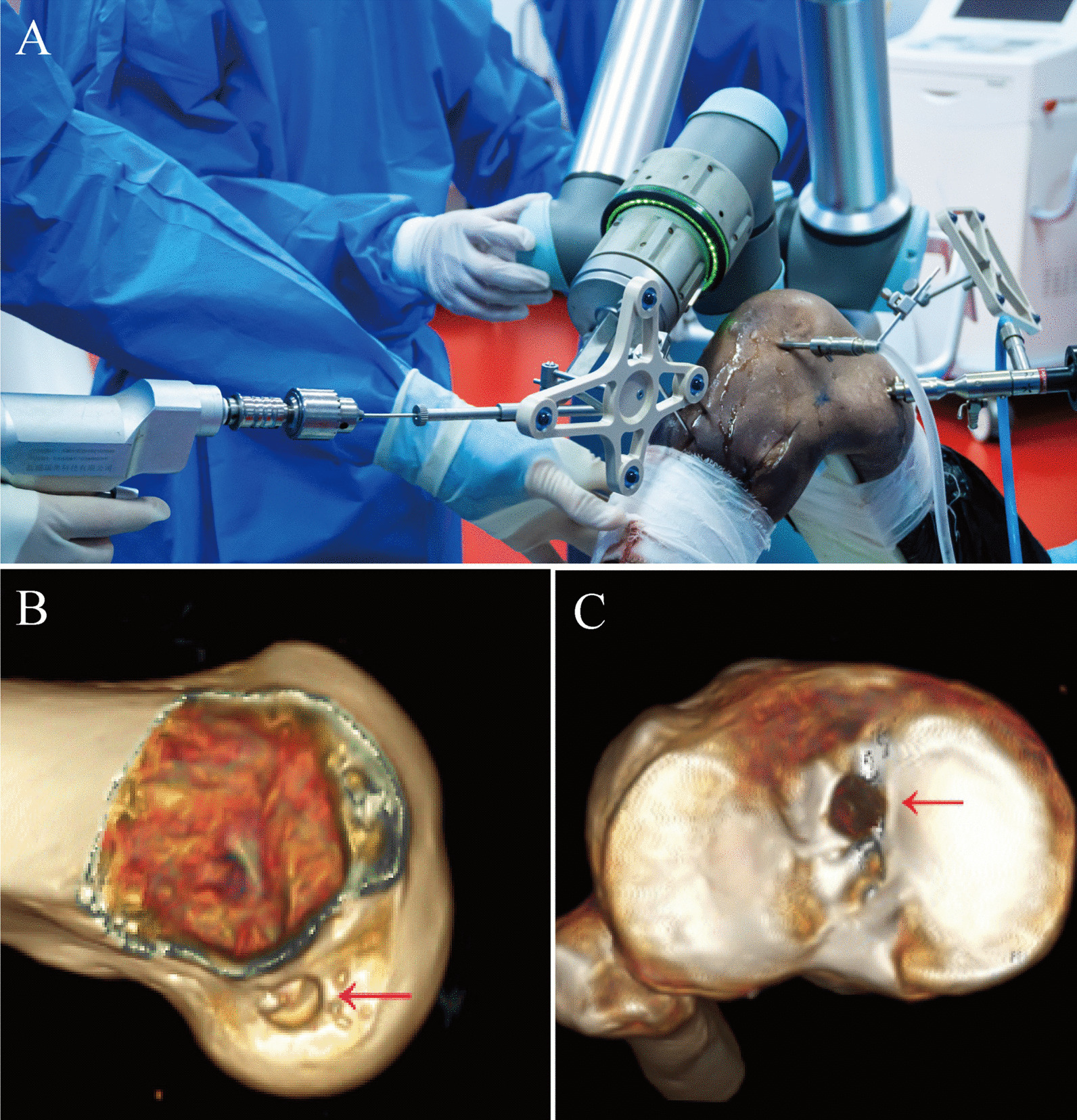
Tunnel location as shown in the arthroscopic field and 3D CT images. **A** After the robot arm completes the positioning step, the doctor drills the bone tunnel; **B** the location of the intra-articular opening of the femoral tunnel on 3D CT; **C** the location of the intra-articular opening of the tibial tunnel on 3D CT

Next, the tibial tunnel was drilled. Similar to the above procedure, the surgeon selected the ACL tibial tunnel on the workstation, activated the robotic arm to achieve automatic navigation, and drilled the ACL tibial tunnel with a Kirschner wire and an 8 mm tibial drill. Figure 3A shows the tibial tunnel drilled by the surgeon with the robot arm.

### Traditional arthroscopic ACL reconstruction

The patient was in the supine position with the knee in 90° flexion. During the operation, arthroscopic exploration of the knee joint cavity and debridement of the ACL stump were routinely performed. According to the surgeon's personal experience, bony landmarks were used to determine the locations of the ACL femoral and tibial tunnel openings. Traditional ACL femoral and tibial locators (Acufex, Smith & Nephew, USA) were used to determine the location of ACL insertion. Finally, Kirschner wires and 8 mm diameter bone tunnel drills were used to drill the bone tunnel.

### Postoperative measurement

After bone tunnel drilling, 3D CT was performed to evaluate the position of the tunnels. As shown in Fig. 3B, C, the location of the tunnel is indicated by the central point of the tunnel opening in the joint. The quadrant method was used to measure the position of the femoral and tibial tunnels [26, 27]. Rectangular coordinate systems were created on the medial aspect of the lateral femoral condyle and on the tibial plateau to calculate the coordinates of the tunnels and accurately represent the location of the tunnels. The position of the femoral and tibial insertion of the ACL was determined using the studies of Anagha et al. [28] and Pisit et al. [29], respectively.

**Fig. 4:**
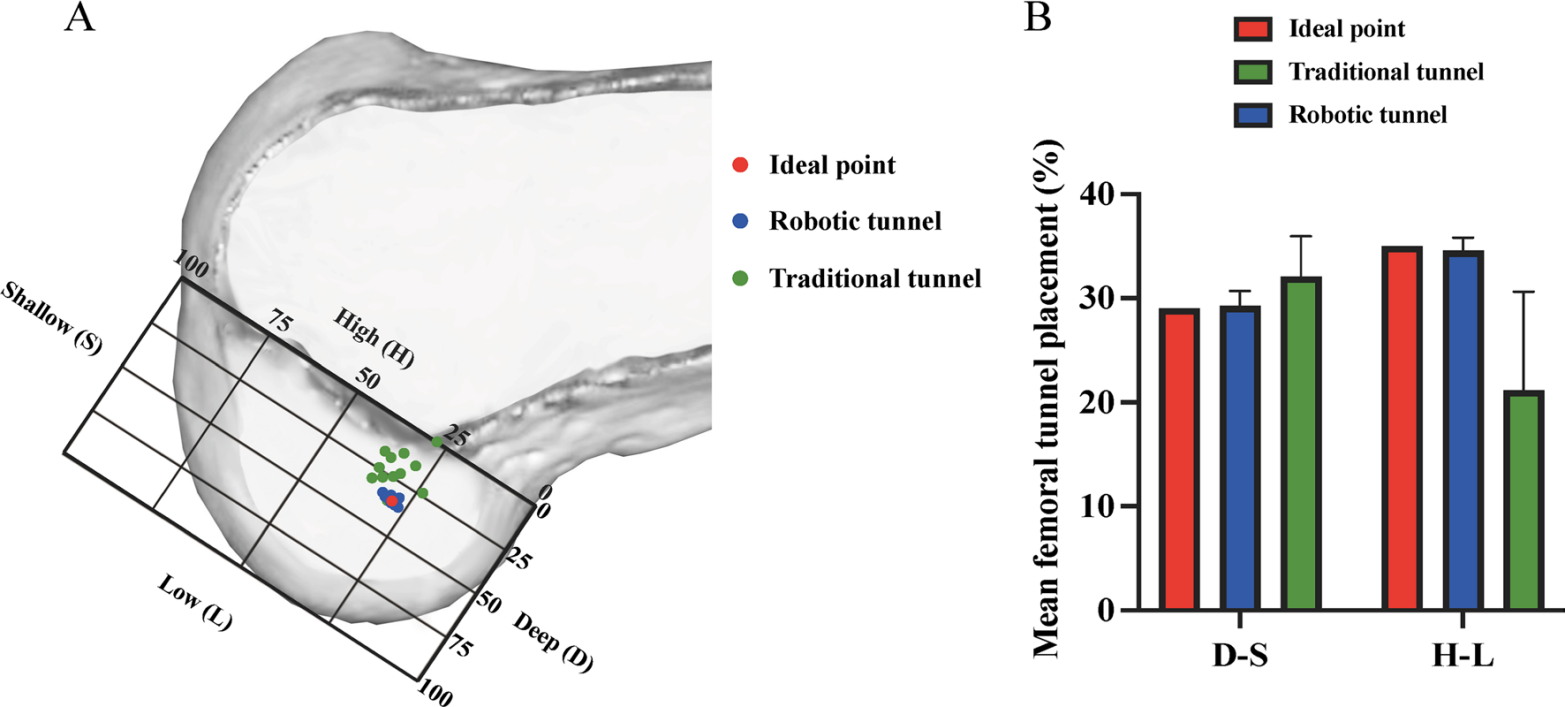
**A** tunnel positions in the femur. The green points represent the tunnel location for traditional surgery, the blue points represent the tunnel location for robotic surgery, and the red points represent the ACL insertion as described in the literature; **B** the mean femoral tunnel position

For the femur, as shown in Fig. 4A, a rectangle was established below Blumensaat’s line, and one edge representing the total sagittal diameter of the lateral femoral condyle of the rectangle was located on Blumensaat’s line. The other edge representing the height of the intercondylar notch is tangential to the posterior margin of the lateral femoral condyle. The position of the femoral tunnel is indicated by the percentage in the deep-shallow (D–S) direction and in the high-low (H–L) direction.

**Fig. 5:**
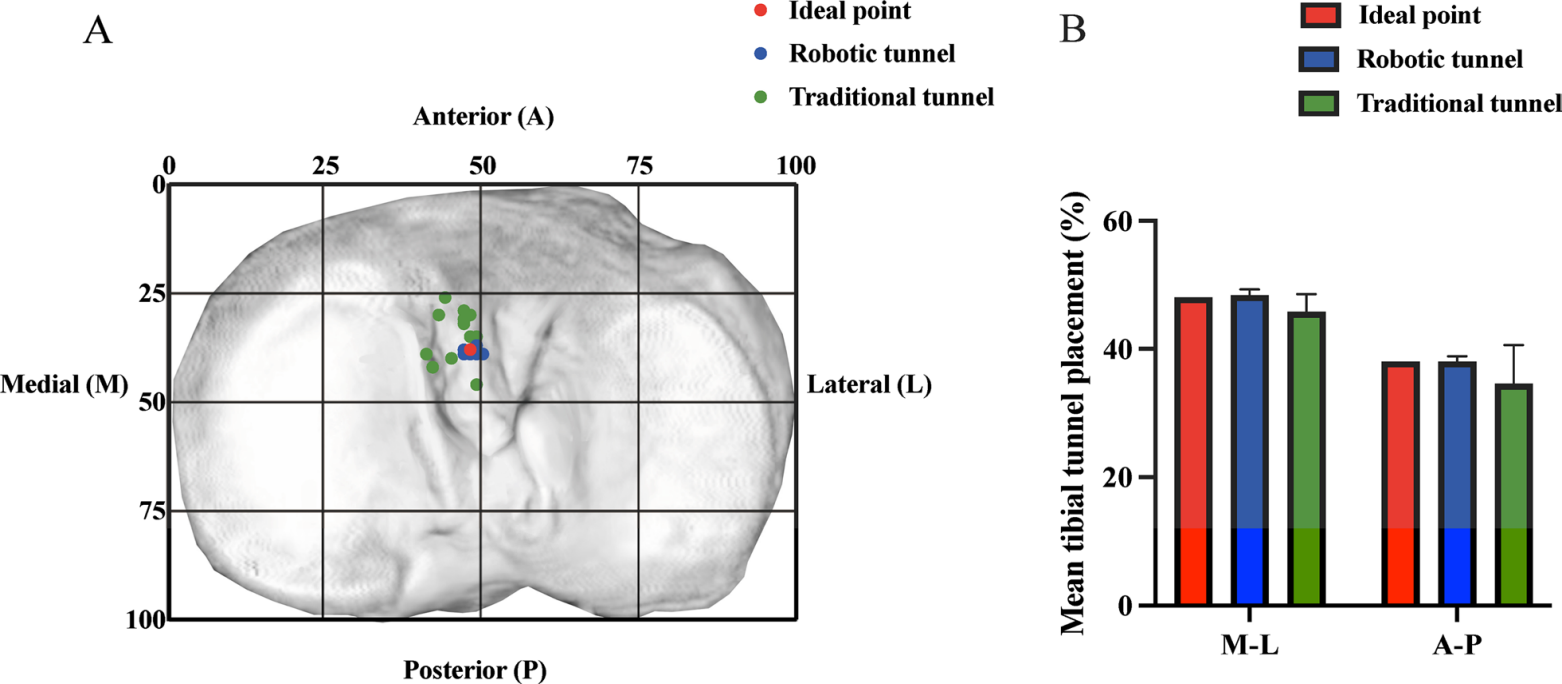
**A** Tunnel positions in the tibia. Green points represent the tunnel location for traditional surgery, blue points represent the tunnel location for robotic surgery, and the red point represents the ACL insertion as described in the literature. **B** The mean tibial tunnel position

For the tibia, as shown in Fig. 5A, a rectangle was built on the tibial plateau with one edge which was tangent to the medial edge of the tibial plateau, parallel to the anteroposterior diameter of the tibial plateau, and the length represented the anteroposterior diameter of the tibial plateau. The other side was tangential to the anterior edge of the tibial plateau, and its length was the mediolateral diameter of the tibial plateau. The position of the tibial tunnel was indicated by the percentage in the medial–lateral (M–L) and the anterior–posterior (A–P) directions.

The error in the bone tunnel position was calculated as the absolute value of the difference between the actual bone tunnel position and the anatomical position of the ACL, expressed as a percentage.

### Statistical analysis

Based on similar previous studies [30], a sample size of 12 knees per group was considered adequate. The Shapiro–Wilk test was used to test the normality of the data. Normally distributed data are expressed as the mean ± standard deviation (SD), and a t test was used for comparisons between groups. Data that did not follow the normal distribution were expressed as median and interquartile range, and comparison between groups was performed using the Mann–Whitney U nonparametric test. SPSS version 27.0 (IBM Corp., Armonk, N.Y., USA) software was used for data analysis. Statistical significance was set at *p* < 0.05.

## Results

Table 1 describes the basic information of the patients' knees. In this experiment, the age of the knee and the duration of surgery were significantly greater in the robotic group than in the conventional group (*p* < 0.001).

**Table 1:** Demographic parameters of the knees and duration of surgery

	Traditional surgery	Robot surgery	*p* value
Laterality			
Left	6	8	
Right	6	4	
Gender			
Female	7	7	
Male	5	5	
Age (years)	34 ± 6.7	70.9 ± 6.0	** < 0.001**
Duration of surgery (min)	27.3 ± 2.7	42.0 ± 2.8	** < 0.001**

Bold values indicate statistically significant differences between groups (*p* < 0.05)

Table 2 summarizes the location information of the bone tunnel. There was no significant difference between the actual bone tunnel position and the insertion position of the ACL in each direction in the robot group (*p* > 0.05), while the bone tunnel position in the traditional surgery group was significantly different from the robot surgery and insertion position in the D–S, H–L and M–L directions (*p* < 0.05). In the A–P direction, the tunnel positions of traditional surgery were not significantly different from either the tunnel positions of robotic surgery or the anatomical positions (*p* > 0.05). The bone tunnel position of the robotic surgery is less variable and closer to the insertion position of the ACL. However, the position of the femoral tunnel in traditional surgery is shallower and higher than that of ACL insertion, and the traditional tibial tunnel position is more medial than ACL insertion. The specific locations of the tibial and femoral tunnels are shown in Figs. 4 and 5.

**Table 2:** Positional information of the bone tunnel^*^

	Anatomic tunnel position (%)	Robot planned tunnel position (%)	Robot actual tunnel position (%)	Traditional tunnel position (%)
D–S	29^‡^	28.6 ± 1.8	29.3 ± 1.4^†^	32.1 ± 3.9^†‡^
H–L	35^‡^	34.8 ± 1.4	34.6 ± 1.2^†^	21.2 ± 9.4^†‡^
M–L	48^‡^	48.4 ± 0.9	48.0 ± 0.7^†^	45.8 ± 2.8^†‡^
A–P	38	38.2 ± 1.1	38.1 ± 0.8	34.6 ± 6.0

^*^D–S represents the direction of deep to shallow; H–L represents the direction of high to low; M-L represents the direction of medial to lateral; A-P represents the direction of anterior to posterior

^†^the difference between the position of the bone tunnel in traditional and robotic surgery was significant (*p* < 0.05)

^‡^the difference between the position of the bone tunnel in traditional surgery and the position of the anatomical insertion site position of the ACL was significant (*p* < 0.05)

Table 3 summarizes the errors in the bone tunnel position relative to the anatomical position of the ACL for robotic and traditional surgery. The results showed that there was no significant difference in the error in the M–L direction (*p* = 0.089), but there were significant differences in the errors in the D–S, H–L and A–P directions (*p* < 0.05). The error in the robotic surgery group was smaller than that in the traditional surgery group.

**Table 3:** The difference between the actual bone tunnel position and the anatomical position of the ACL between conventional surgery and robotic surgery^*^

	Robot actual tunnel error (%)	Traditional tunnel error (%)	*p* value
D-S	1.0(0.3, 1.8)	4.5(1.3, 6.8)	**0.006**
H–L	1.0(0.0, 1.0)	12.0(7.8, 20.0)	** < 0.001**
M-L	1.0(0.0, 1.0)	1.0(1.0, 4.8)	0.089
A-P	1.0(0.0, 1.0)	6.5(3.0, 8.0)	** < 0.001**

^*^D-S represents the direction of deep to shallow; H–L represents the direction of high to low; M-L represents the direction of medial to lateral; A-P represents the direction of anterior to posterior

Bold values indicate statistically significant differences between groups (*p* < 0.05)

Robot planned and actual bone tunnel lengths are summarized in Table 4. There was a significant difference between the planned and actual lengths of the tunnels (*p* < 0.001), but the error did not exceed 1.5 mm in any of the tunnels.

**Table 4:** The length of the planned bone tunnel and the actual bone tunnel in the robotic surgery

	Planned tunnel length (mm)	Actual tunnel length (mm)	Error (mm)	*p* value
Femur	40.8 ± 3.1	39.9 ± 3.0	0.9 ± 0.4 (0.3–1.4)	** < 0.001**
Tibia	46.0 ± 5.0	45.0 ± 5.1	1.0 ± 0.4 (0.1–1.5)	** < 0.001**

Bold values indicate statistically significant differences between groups (*p* < 0.05)

## Discussion

The most important finding of this study was that the surgical robot system was able to drill the bone tunnel accurately according to the planned position of the surgeon during the operation, and the bone tunnel position was closer to the ACL insertion position than traditional arthroscopic surgery. According to previous studies, the femoral attachment of the ACL is located, on average, at 29% of the deep-to-shallow distance and 35% of the high-to-low distance along the lateral femoral condyle; the tibial attachment is located at 48% of the medial to lateral distance and 38% of the anterior-to-posterior distance of the tibial plateau [28, 29].

With the development of arthroscopic anatomic ACL reconstruction, although the technology is mature, the surgical instruments are advanced, and there are many bony landmarks that can be referred to; moreover, the actual location of the bone tunnel may still be quite different from the anatomical point [31–33]. However, nonanatomical ACL reconstruction increases the risk of revision surgery [34]. As a new surgical method, robot-assisted orthopedic surgery has been widely used in the fields of spine, joint and trauma surgery, improving the accuracy of surgery and postoperative efficacy. However, there are few reports on surgical robots in the field of sports medicine. ACL reconstruction, the most common operation in sports medicine, requires highly accurate bone tunnel drilling to restore the normal function of the knee joint. In our study, there were two main reasons why robotic surgery was more accurate than traditional surgery: 1. In traditional surgery, the anatomical insertion of the ACL is manually determined by the surgeon (using a locator under arthroscopy visualization), while the stump of the ACL may vary under arthroscopy, and the surgeon cannot fully visualize the positional relationship between the tunnels and the knee joint. However, in robotic surgery, after the ACL insertion is located under arthroscopy, the surgeon adjusts the position of the tunnel through the 3D image of the knee joint in the robot workstation. The adjustment is completed according to the spatial position of the tunnels with respect to the bony landmarks of the femur and tibia, increasing the accuracy and consistency of the tunnel position. The relevant literature has shown that 3D images are helpful for surgeons to complete accurate ACL tunnel localization, and auxiliary tools should be used during ACL reconstruction to reduce tunnel variability [35, 36]. Moreover, it facilitates the surgeons’ design of bone tunnels with appropriate parameters such as the length and angle and reduces the risk of blowing out of the posterior wall. The combination of arthroscopy allows surgeons to locate the remnant of the ACL visually, preventing planning of the tunnel from damaging the posterior cruciate ligament, meniscus, or articular cartilage of the knee, and ensuring drilling safety under direct visualization. 2. The tunnel drilling of traditional surgery involves manual drilling by a surgeon using a traditional handheld locator, which can cause jitter and displacement. In robotic surgery, the robotic arm is used to drill the tunnel, and the robotic arm locks after completing the positioning and navigation. The robot arm has both accuracy and stability that are far better than those of the human hand. It minimizes the position error caused by jitter. Combined with these two advantages, robotic surgery has achieved more accurate results.

Another finding in our study was that there was less variability of the bone tunnel position was less with robotic surgery than with traditional surgery. In our study, the surgeon who performed the procedure had 20 years of experience in arthroscopic surgery. Therefore, even high-volume surgeons are not always able to drill the bone tunnel of the ACL in their planned position, and various methods can be used to enhance the ability of these surgeons to perform ACL bone tunnel placement. This finding is similar to the conclusion of James et al. [37]. Furthermore, arthroscopic ACL reconstruction may be a great challenge for surgeons with low surgical volumes. Erik et.al [38]. found that in ACL reconstruction, the accuracy of bone tunnel positioning improves with the cumulative number of operations, and it takes approximately 100 operations are required before the tunnel position can be significantly improved. The emergence of the robotic approach may provide surgeons with simpler and more convenient surgical tools.

There are no established criteria for the length of the femoral and tibial tunnels in ACL reconstruction. However, a tunnel length of less than 25 mm may affect postoperative healing due to an insufficient tendon-bone contact area [39–41]. The robotic system used in this study can adjust the length of the femoral and tibial tunnels during the course of intraoperative planning, thus avoiding bone tunnels that are too short. The actual lengths of the femoral and tibial tunnels drilled by the robot were 39.9 mm and 45.0 mm, respectively, and the lengths of the bone tunnels were sufficient. In addition, all the errors were less than 1.5 mm compared with the planned bone tunnel length.

The limitations of this study should be acknowledged. First, knees that underwent conventional ACL reconstruction were younger than that underwent robotic surgery, possibly leading to age-related differences in the anatomy of the knee. However, we used preoperative MRI to exclude knees with severe joint disease and percentage values to standardize the location of the bone tunnels, thereby reducing the influence of age on the results. Second, the same surgeon performed all surgical procedures, not allowing representation of all levels of the surgeon population. However, this was done to exclude the influence of different surgeons on the results. Third, the main reasons for the long operation time in this study were as follows: 1. The acquisition and registration of intraoperative 3D images, which ensures the accuracy of the robotic surgery, may take a few minutes longer in actual operation. 2. In this study, the surgeons were not skilled enough in robotic surgery. When surgeons master the surgical techniques, the operation time is expected to be greatly reduced. Finally, we only compared the differences in accuracy between the robot and conventional surgery methods and did not compare postoperative knee function. Further studies are needed to provide more clinical evidence for the application of robots in the future.

## Conclusions

Robot-assisted ACL reconstruction can ensure consistency in the drilling of the bone tunnel and ACL insertion and is more accurate than traditional surgery. Robots are expected to become a powerful tool for ACL reconstruction surgery.

## Conflict of interest

The authors declare that they have no conflicts of interest.

## Data Availability

The data that support the findings of this study are available from the corresponding author upon reasonable request.
